# The Differential Expression of EphB2 and EphB4 Receptor Kinases in Normal Bladder and in Transitional Cell Carcinoma of the Bladder

**DOI:** 10.1371/journal.pone.0105326

**Published:** 2014-08-22

**Authors:** Xiuqing Li, Wesley W. Choi, Rui Yan, Haiyang Yu, Valery Krasnoperov, S. Ram Kumar, Anne Schuckman, David J. Klumpp, Chong-Xian Pan, David Quinn, Inderbir S. Gill, Parkash S. Gill, Ren Liu

**Affiliations:** 1 Department of Medicine, University of Southern California Keck School of Medicine, Los Angeles, California, United States of America; 2 Department of Urology, University of Southern California Keck School of Medicine, Los Angeles, California, United States of America; 3 Department of Surgery, Central South University National Hepatobiliary & Enteric Surgery Research Center, Changsha, Hunan, People's Republic of China; 4 VasGene Therapeutics Incorporated, Los Angeles, California, United States of America; 5 Department of Surgery, University of Southern California Keck School of Medicine, Los Angeles, California, United States of America; 6 Department of Urology, Northwestern University Feinberg School of Medicine, Chicago, Illinois, United States of America; 7 Department of Internal Medicine, University of California at Davis Comprehensive Cancer, Sacramento, California, United States of America; University of Central Florida, United States of America

## Abstract

Effective treatment of transitional cell carcinoma (TCC) of the bladder requires early diagnosis. Identifying novel molecular markers in TCC would guide the development of diagnostic and therapeutic targets. Ephrins mediate signals via tyrosine kinase activity that modulates diverse physiologic and developmental processes, and ephrins are increasingly implicated in carcinogenesis. The aim of our study was to examine the differential regulation of EphB4 and EphB2 in normal bladder and in TCC of the bladder in 40 patients undergoing radical cystectomy for curative intent. Immunostaining and Western blotting revealed that normal urothelium expresses EphB2 (20 of 24 cases, 83% of the time) not EphB4 (0 of 24 cases, 0%). In sharp contrast, TCC specimens show loss of EphB2 expression (0 of 34 cases, 0%) and gain of EphB4 expression (32 of 34, 94%). Furthermore, EphB4 signal strength statistically correlated with higher tumor stage, and trended toward the presence of carcinoma in situ (CIS). These results are confirmed by analysis of normal urothelial and tumor cell lines. EphB2 is not a survival factor in normal urothelium, while EphB4 is a survival factor in TCC. Treatment of bladder tumor xenograft with an EphB4 inhibitor sEphB4-HSA leads to 62% tumor regression and complete remission when combined with Bevacizumab. Furthermore, tissue analysis revealed that sEphB4-HSA led to increased apoptosis, decreased proliferation, and reduced vessel density, implicating direct tumor cell targeting as well as anti-angiogenesis effect. In summary loss of EphB2 and gain of EphB4 expression represents an inflection point in the development, growth and possibly progression of TCC. Therapeutic compounds targeting EphB4 have potential for diagnosing and treating TCC.

## Introduction

Bladder cancer is estimated to afflict over 74,000 people in the United States each year and results in 15,000 deaths [1]. The hallmark of bladder cancer is its propensity for recurrence and progression. Bladder cancer has the highest local recurrence rate of any malignancy [2]. It is estimated that as many as 75% of superficial tumors will reoccur and nearly 30% of these recurrences will progress to more invasive and lethal cancers. Cystoscopy and cytology are currently the most frequent modalities employed to determine the presence of TCC. However, cystoscopy can be uncomfortable to the patient, expensive, and often inconclusive, while cytology can miss up to 50% of low grade bladder lesions [2].

Transitional cell carcinoma (TCC) represents 90% of all bladder cancers in the US, and remains a “surgical disease”- the best outcomes are obtained early in the disease process when complete surgical excision is possible [3]. Despite an overall 5 year survival rate of 82%, the five year rate for localized TCC is 94%, while only 6% for metastatic disease [4]. Thus, there is a clear need for identifying both novel diagnostic tools and more effective targets for novel systemic therapies.

EphB4 is a member of the largest known family of receptor protein tyrosine kinases and plays important diverse roles in pattern formation, axon guidance, angiogenesis, vascular network assembly, and cloacal development [5]–[8]. EphB4 is normally expressed on venous endothelial cells, while its exclusive ligand, EphrinB2, is expressed on arterial endothelial cells. Interaction between EphB4 and EphrinB2 induces bidirectional signaling to exact changes in essential for defining the boundaries between arterial and venous domains [6]. EphB4 and EphrinB2 are expressed in adult life and are required for the development/maturation of newly forming vessels only, and thus represent targets for modulation of angiogenesis including cancer [9]. Over-expression of EphB4 has been observed in a number of different tumors, including prostate, breast, head and neck, uterine and mesothelioma [10]–[18]. Previously, we showed that EphB4 was over-expressed in bladder cancer in a small number of cases [19]. Furthermore we showed that EphB4 provided survival advantage to bladder cancer cells in vitro and in vivo.

EphB2, also a member of the Eph family of receptor protein tyrosine kinases, has been extensively studied in colon cancer. In the proliferative crypts of the colon, EphB2 acts to control cell compartmentalization [20]. Loss of EphB2 expression has been shown to correlate with more advanced colorectal cancer, poorer differentiation, and poorer overall survival [21]. EphB2 also plays an important role in familial prostate cancer. Loss of function mutations in the EphB2 gene have been shown to be associated with prostate cancer risk in African American men with positive family histories [22]. Of relevance to genitourinary tract development, EphB2 mutations result in hypospadias and cloaca in mouse models which implies a role for EphB2 in the midline fusion of the anus and lower urinary tract during development [23]. The role of EphB2 in the bladder has not been studied. We hypothesized that EphB2 is expressed in normal bladder and lost in bladder cancer, similar to the observation in colon cancer, while induction of EphB4 in bladder cancer provides survival advantage. If this is the case, targeting EphB4 would kill tumor cells and spare normal bladder and thus lack toxicity.

To elucidate the roles of EphB4 and EphB2 in TCC of the bladder, we examined the expression of EphB4 and EphB2 in normal and TCC surgical bladder specimens, and also in bladder cancer cell and immortalized normal urothelial cell lines. We found EphB4 is consistently over-expressed while EphB2 expression is predominantly absent in bladder cancer. In sharp contrast, high EphB2 and very low EphB4 expression are observed in normal urothelium. An inhibitor of EphB4 in a bladder tumor xenograft model significantly inhibited tumor cell proliferation and angiogenesis, and also induced apoptosis and overall tumor regression. These results indicate that EphB4 is a potential therapeutic target in bladder cancer.

## Materials and Methods

### Ethics Statement

Human tissues were collected under the University of Southern California (USC) Institutional Review Board approval and signed informed patient consent. All animal procedures were approved by USC Institutional Animal Care and Use Committee and performed in accordance with the Animal Welfare Act regulations.

### Reagents

Media and fetal bovine serum were from Invitrogen (Carlsbad, CA). Monoclonal EphB4 antibodies used in immunohistochemistry and immunoprecipitation (Clone #131) and Western blotting (Clone #265) were from VasGene Inc. (Los Angeles, CA). EphB2 antibody was from R&D systems (Minneapolis, MN). Phospho-Tyrosine antibody (Clone 4G10) was from Millipore (Billerica, MA). CD31 antibody was from BD Biosciences (San Jose, CA). Ki67 antibody was from Abcam (Cambridge, MA). Phosphorylated S6 (Ser235/236) antibody was from Cell Signaling (Danvers, MA). Horse radish peroxidase (HRP) conjugated secondary antibodies were from Rockland (Gilbertsville, PA).

### Cell lines and culture

5637 bladder cancer cells were obtained from Dr. Peter A Jones (USC) and cultured in Roswell Park Memorial Institute (RPMI)-1640 medium containing 10% fetal bovine serum, 5 mmol/L L-glutamine, and penicillin/streptomycin. PD07I normal bladder urothelium cells were from Dr. Klumpp (Northwestern University, Chicago, IL) and cultured in EpiLife Medium (Cascade Biologics) supplemented with HKGS (Cascade Biologics) and PSA (Cascade Biologics) [24].

### Human tissues

A total of 40 paired specimens of bladder cancer were obtained from radical cystectomy specimens undertaken with curative intent. Adjacent normal tissue was also collected when possible. Human tissues were collected under the USC Institutional Review Board approval and signed informed patient consent. Tissues were analyzed and graded by a blinded reviewer.

### Western blot

Cultured cells and tumor samples were lysed with Cell Lysis Buffer (GeneHunter, Basgvukke, TN) supplemented with protease inhibitor cocktail (Pierce, Rockford, IL). Protein concentration was measured using the DC reagent system (Bio-Rad, Hercules, CA) and 20 ug of protein lysate was run on a 4–20% Tris-glycine gradient gel (Bio-Rad) and transferred to a nitrocellulose membrane (Bio-Rad). The membrane was blocked with 5% nonfat milk, incubated with primary antibody at 4°C for overnight and then appropriate horse radish peroxidase conjugated secondary antibodies at room temperature for 1 h. SuperSignal West Femto Maximum Sensitivity Substrate (Pierce, Rockford IL) was used for signal development.

### Immunostaining

Human normal bladder and bladder cancer specimens were obtained under Institutional Review Board-approved protocols. Sections (7 µm) of fresh frozen human bladder tumor tissues were fixed in 4% paraformaldehyde and blocked with SuperBlock blocking buffer (Pierce, Rockford, IL). Sections were incubated with primary antibody overnight at 4°C and appropriate secondary antibody for 1 hour at room temperature. Antibody binding was localized with Vectastain ABC staining kit (Vector Laboratories, Burlingame, CA) according to the manufacturer's instructions. For H&E staining, sections were counterstained with Harris hematoxylin for 45 s, dehydrated, and mounted in xylene. For immunofluorescence, secondary antibody incubation was followed by incubation for 30 minutes at room temperature with 2 µg/ml fluorescein isothiocyanate-labeled avidin (Vector) and nuclei were counterstained with 4',6-diamidino-2-phenylindole (DAPI). Routine negative controls included omission of primary or secondary antibody and substitution of isotope control IgG for primary antibody. EphB4 signal strength was assigned to each specimen by a blinded reviewer as “none,” “+”, “++”, “+++”, or “++++”. Weak signal strength included all stains graded as “+”, or “++”. Strong signal strength included those stained and recorded as “+++” or “++++”. In xenograft tumor analysis, NIH ImageJ software was used for signal quantification. At least 3 pictures from each analysis were used for quantification.

### siRNAs

EphB4 siRNA (sequence was 5'-CCGGGAAGGUGAAUGUCAA-3') and EphB2 siRNA (Hs_EPHB2_10 HP Validated siRNA, sequence undisclosed) were synthesized from Qiagen (Valencia, CA). Lipofectamine 2000 (Invitrogen, Carlsbad, CA) was used for siRNA transfection following manufacturer's instruction.

### Immunoprecipitation

Freshly frozen tumors were homogenized in Homogenization Buffer (25 mM Tris (pH 8.0), 150 mM NaCl, 2 mM sodium vanadate, 1 mM sodium fluoride, and 1x proteinase inhibitor cocktail (Thermo Fisher, Waltham, MA)), and then membrane proteins were solubilized in Homogenization Buffer supplemented with 1% Triton X-100. Tumor lysates were incubated with protein G beads and EphB4 specific antibody MAb131 overnight at 4°C and the immunoprecipitated EphB4 was analyzed by Western Blot as described above.

### Cell Viability Assay

5637 cells and PD07I cells were seeded in 48-well plates at a density of 1×10^4^ cells/well in a total volume of 500 µl. Medium was changed after cells were attached, and triplicate samples were treated as described in Results. Cell viability was assessed using 3-(4,5-dimethylthiazol-2-yl)-2,5-diphenyltetrazolium bromide (MTT) after treatment with siRNA to EphB2 and EphB4 as described previously [25]. Alternatively, cell apoptosis was analyzed with Annexin V Apoptosis Quantitation Kit (Biotium, Hayward, CA) following the manufacturer's instructions.

### *In vivo* tumor growth studies

The procedure of tumor xenograft study was described previously [18]. Briefly, athymic BALB/c mice were injected with 5×10^6^ 5637 bladder cancer cells in the flank. When tumor sizes reached 250 mm^3^, mice were grouped (n = 8) and treated (3 times weekly) with intraperitoneal (i.p.) injection of PBS (control), sEphB4-HSA (20 mg/kg), Bevacizumab (20 mg/kg), or a combination of sEphB4-HSA and Bevacizumab. Tumor size was calculated with the formula V = 0.52ab^2^, where V is the tumor volume and a and b are the longest and shortest dimensions of a palpable tumor. All animal procedures were approved by USC Institutional Animal Care and Use Committee and performed in accordance with the Animal Welfare Act regulations.

### Statistical analyses

Differences in EphB2 and EphB4 staining in normal and in tumor, as well as EphB4 staining intensity at various cancer stages were analyzed with t-test. Significance was set at *P*<0.05. Two tailed, unpaired student T test was used in the xenograft study and tumor staining analysis.

## Results

### Normal urothelium expresses EphB2 but not EphB4

**Figure 1:**
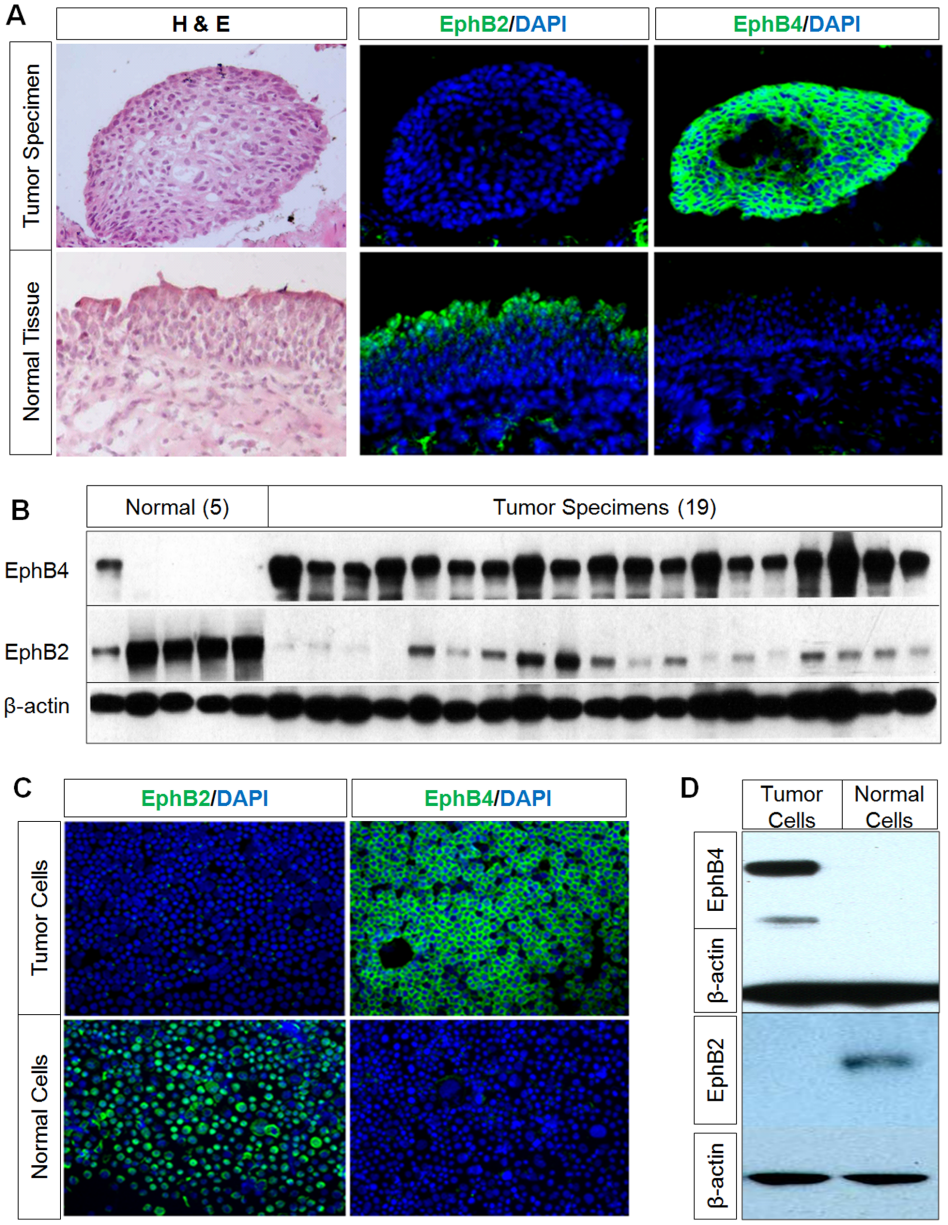
Reciprocal expression of EphB4 and EphB2 in bladder tumor and normal bladder cells. A, Representative immunostaining on tumor and normal urothelium obtained from the same patient during cystectomy along with H&E stains showing EphB4 positivity in tumor specimens but not in normal tissue. In contrast there is EphB2 staining in normal tissue, but not in tumor specimens. Nuclei were counter-stained with DAPI. B, Western blot analysis of EphB4 and EphB2 in five normal bladder and 19 bladder tumor tissues shows high EphB4 expression in tumor but low or no expression in normal tissue and reciprocal expression pattern of EphB2. β-actin immunoblotting shows equal protein loading. C, Immunofluorescence staining of bladder cancer cell 5637 and normal urothelial cell PD07I also show strong EphB4 and absent EphB2 staining in tumor cells. There is strong EphB2 and absent EphB4 staining in normal cells. D, Western blot analysis shows EphB4 in tumor cell line 5637 but not in the normal cell line PD07I, whereas EphB2 is present in the normal cell line but not in the tumor cell line.

Normal urothelium and adjacent tumor specimen were obtained from 40 patients undergoing radical cystectomy (Table S1 in File S1). Histological examination showed that only 34 of the 40 specimens contained tumor. Thus all studies related to expression, and analysis of EphB receptors is limited to 34 samples. Normal bladder tissue was available on 24 of the 40 cases, and only 18 of the 24 had matched tumor and normal tissue for analysis. We first characterized the expression of EphB2 and EphB4 in normal urothelium. EphB2 and EphB4 expression were characterized by immunofluorescence in all 24 of the normal urothelial specimens obtained at the time of radical cystectomy. Of the normal urothelial specimens, 20/24 (83%) stained positive for EphB2, while 0/24 (0%) stained positive for EphB4 (Table 1 and Figure 1A). Western blot analysis confirmed the absence of the EphB4 receptor and high EphB2 expression in normal urothelium in a subset of the cases studied (Figure 1B). There is one normal urothelium sample showing weak EphB4 expression and relatively low EphB2 expression which is likely related to "field effect". Immunofluorescent staining of this adjacent tissue had atypical morphology and faint positivity for EphB4 suggesting that this normal adjacent tissue might have been predisposed to oncogenic transformation. We then examined the immortalized normal urothelial cell line (PD07I) and noted strong EphB2 immunofluorescence staining without EphB4 staining (Figure 1C). Western blot analysis of PD07I cells confirmed the presence of the EphB2 receptor and the absence of EphB4 in the immortalized normal urothelial cell line (Figure 1D).

**Table 1:** Summary of immunofluorescence staining analysis of EphB4 and EphB2 expression in normal and tumor urothelium specimens.

			Positive number (percentage)	
Urothelium specimens		Total number	EphB2+	EphB4+
Overall	Normal	24	20 (83)*	0 (0)
	Tumor	34	0 (0)	32 (92)*
Matched samples	Normal	18	14 (78)*	0 (0)
	Tumor	18	0 (0)	16 (89)*

*P<0.001, determined by two sample t-test.

### EphB2 expression is lost and EphB4 is induced in TCC

Next, EphB2 and EphB4 expression was also characterized by immunofluorescence in all 34 of the TCC specimens obtained at surgery. Of the TCC specimens, 0/34 (0%) stained positive for EphB2, while 32/34 (94%) stained positive for EphB4 (Table 1 and Figure 1A). Western blot analysis also confirmed markedly increased EphB4 expression in tumor specimens. EphB2 expression in the tumor samples had no or low levels of EphB2 compared to the normal bladder. The discrepancy in Western blot compared to immunofluorescence analysis suggests that Western blot is more sensitive than immunofluorescence or some tumor tissues used for Western blot were contaminated by normal cells thus giving a low level EphB2 signal. The switch from EphB2 to EphB4 from normal bladder to tumor remains a prominent finding based on two orthogonal methods. EphB4 expression is confirmed in bladder cancer cell lines including 5637 demonstrating strong EphB4 staining with little or no EphB2 protein expression by Western Blot and immunostaining (Figure 1 C, D).

### The matched cases of normal and tumor specimens from the same patient also show similar results of EphB2 and EphB4 expression

Of the 34 tumor samples there were 18 matched cases where both normal and tumor specimens were histologically confirmed. We examined this subset of data to ensure the generalizability of the overall data. Of the matched cases, 14 of the 18 (78%) normal urothelial samples showed an EphB2 signal, while 0 of 18 (0%) stained for EphB4. Furthermore, 16 of the 18 tumor (89%) specimens showed an EphB4 signal, while 0 of 18 (0%) stained for EphB2 (Table 1). Representative staining pictures are shown in Figure S1 in File S1. This subset analysis of matched specimens revealed results similar to the overall results.

### EphB4 signal strength statistically correlates with higher tumor stage, and trends toward presence of carcinoma in situ (CIS)

The clinical and pathologic characteristics of the 34 patients in which tumor specimens were received were also examined and correlated with EphB4 signal strength. The same analysis was not performed for EphB2 because EphB2 expression is lost in tumor. We found that strong EphB4 signal strength was statistically associated with those tumors with invasive disease (pathologic stages p2 and higher). 26 of the 30 (87%) tumor specimens with invasive disease stained with strong signal strength (*P*<0.001, Table S2 in File S1), while only 2 of the 4 (50%) tumor specimens with superficial disease stained with strong signal strength.

We also observed trend towards higher EphB4 signal in tumors with accompanying carcinoma-in-situ. 21 of the 24 (88%) CIS positive tumor specimens stained with strong EphB4 signal (*P*<0.001, Table S2 in File S1), while only 7 of the 10 (70%) CIS negative tumor specimens stained with strong signal strength (not significant, Table S2 in File S1). Larger number of cases are needed to further investigate these findings.

### EphB2 is not a survival factor in normal urothelium, while EphB4 is a survival factor in TCC

**Figure 2:**
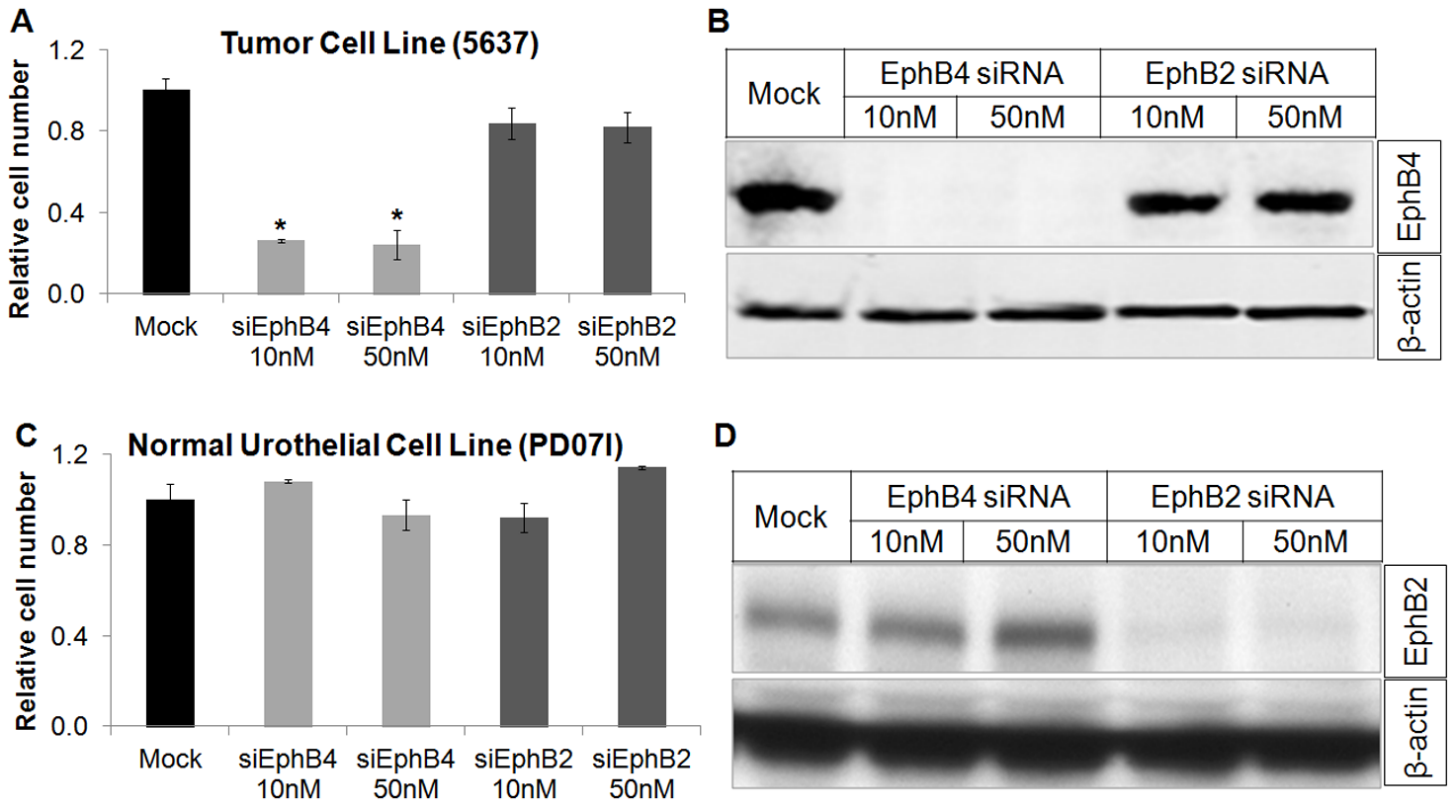
EphB4 provides survival advantage to bladder tumor cells. A, Tumor cell line expressing EphB4 (5637) was treated with various doses of EphB2 or EphB4 siRNA and viable cell number was determined using MTT assay. Cells were treated for 48 hrs. Data are presented as mean ± standard deviation (n = 3). Knockdown of EphB4 but not EphB2 significantly reduced viable tumor cell number. Asterisk indicates *P*<0.002, as determined by an unpaired 2-tail student T-test. B, Protein lysates were examined for EphB4 levels. EphB4 expression was effectively knocked down with EphB4 siRNA (β-actin as loading control). C, MTT assay of the normal urothelial cell line PD07I showed no effect on cell viability with EphB2 or EphB4 knockdown with varying siRNA dosages. D, Protein lysates were measured by Western blot analysis, showing almost complete abrogation of EphB2 protein with EphB2 siRNA.

Previously, we demonstrated that EphB4 is a cell survival factor in the TCC cell line 5637 (14), which has high EphB4 and no EphB2 expression (Figure 1C, D). MTT assay determined that knockdown of EphB4 with siRNA reduced viable cell number of 5637 cells, whereas knockdown of EphB2 had no effect (Figure 2A, B). Annexin V flow cytometry analysis further showed EphB4 siRNA induced 5637 apoptosis whereas EphB2 siRNA had no effect (Figure S2A in File S1). We next used normal urothelial cell PD07I which expresses EphB2 but not EphB4 (Figure 1C, D) to determine if EphB2 is a cell survival factor in normal urothelium. EphB2 specific siRNA did not affect cell growth and viability at any of the two concentrations studied (10 and 50 nM) (Figure 2 C, D), as well as apoptosis (Figure S2B in File S1).

### *In Vivo* efficacy of sEphB4-HSA alone and in combination with bevacizumab

**Figure 3:**
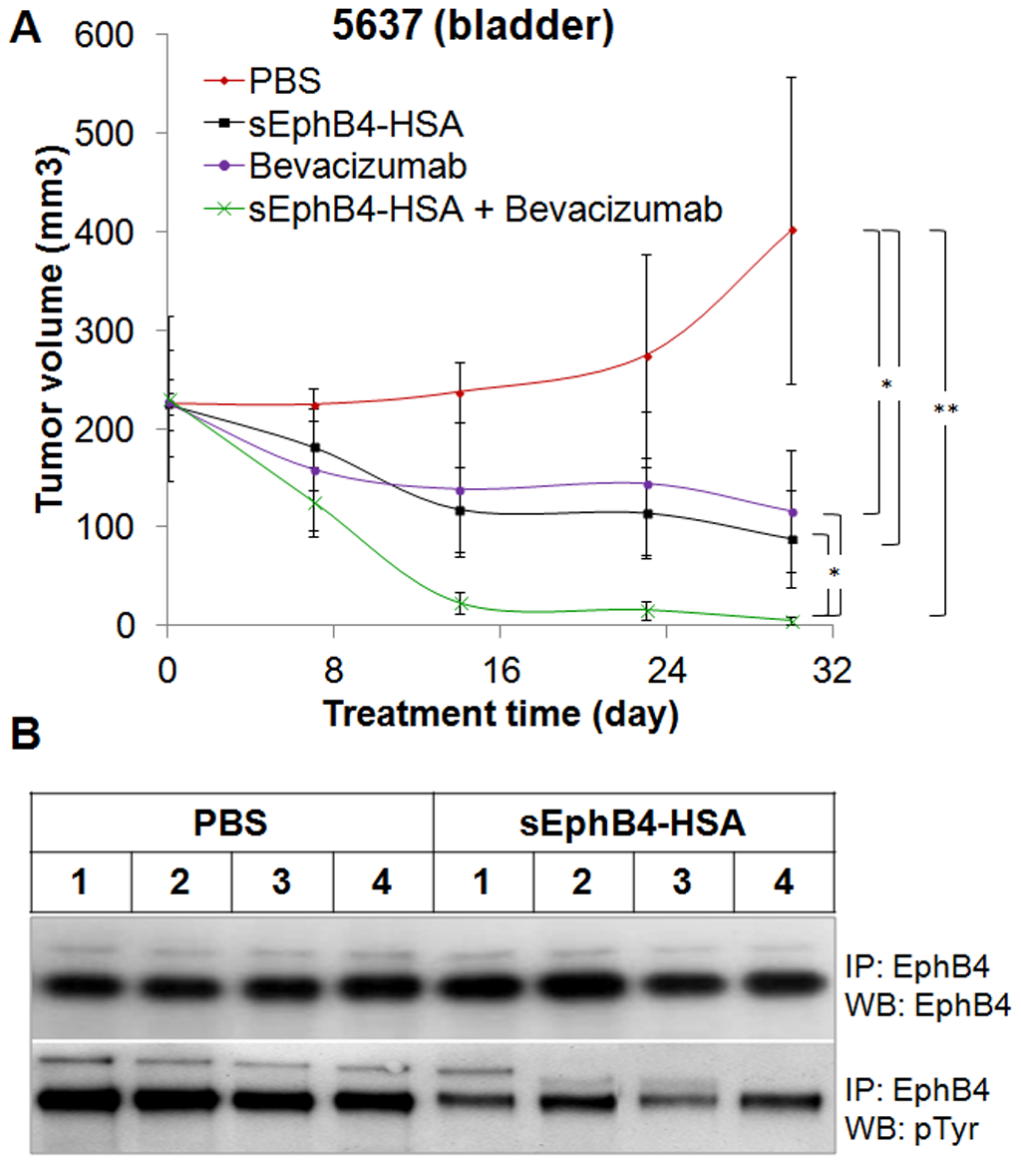
*In vivo* efficacy of sEphB4-HSA combined with Bevacizumab. A, 5637 tumors were treated with sEphB4-HSA alone (20 mg/kg, 3 times a week), Bevacizamab alone (20 mg/kg, 3 times a week), or sEphB4-HSA combined with Bevacizamab. PBS was used as control. Data are presented as mean ± standard deviation. Student *t*-test (2 tails, unpaired) was used to calculate P value: *, P<0.05; **, P<0.01. B, tumors harvested from the xenograft study were lysed for EphB4 immunoprecipitation, followed by immunoblotting using EphB4 and phopho-tyrosine antibodies. sEphB4-HSA treatment significantly reduced EphB4 tyrosine phosphorylation *in vivo*.

**Figure 4:**
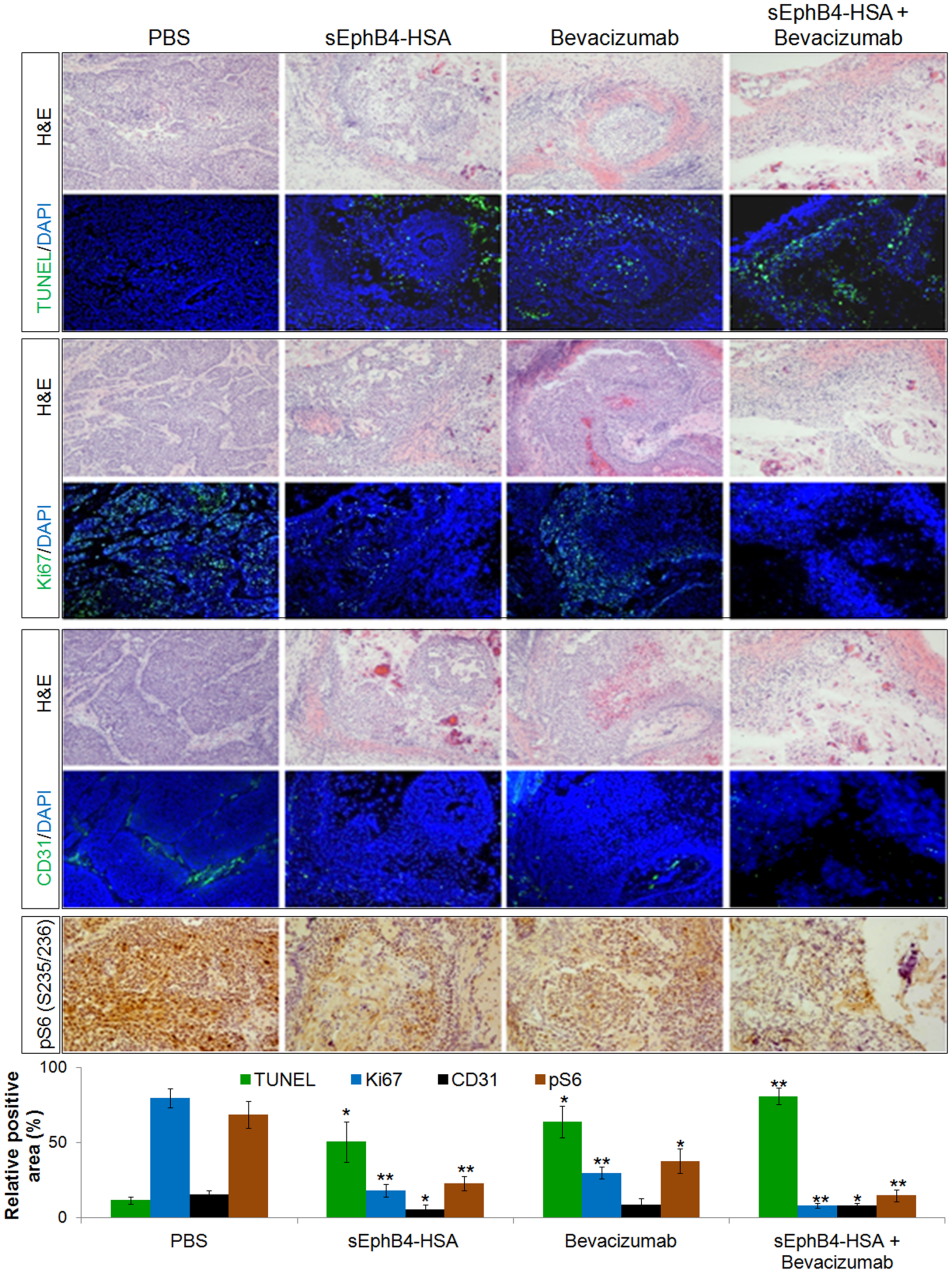
Combinatorial effect of sEphB4-HSA and Bevacizamab on tumor vasculature and tumor cells. 5637 tumors shown in Figure 3 were harvested for immunofluorescence analysis. Tumor vessel density was evaluated by CD31 staining. Cell proliferative index was evaluated by ki67 staining and apoptosis was assessed with TUNEL staining. PI3K signaling was analyzed with phosphorylated S6 staining. At least 3 pictures from each analysis were used for quantification. Data are presented as mean ± standard deviation. Student *t*-test (2 tails, unpaired) was used to calculate P value: *, P<0.05; **, P<0.01.

sEphB4 is the extracellular domain of EphB4 that blocks EphB4-Ephrin-B2 bi-directional signalling thus acts as an EphB4 inhibitor [26]. sEphB4-HSA is a fusion protein with human serum albumin on the C-terminus of sEphB4, which has improved half life and delivery [18]. Both sEphB4 and sEphB4-HSA have shown anti-tumor activities in many tumor models [18], [26]–[30]. Here, we studied the anti-tumor activity of sEphB4-HSA in bladder cancer xenograft models using the human bladder cancer cell line 5637 that has robust over-expression of EphB4. sEphB4-HSA alone showed very good efficacy. Tumors treated with sEphB4-HSA had a 62% regression from the starting tumor volume (Figure 3A). sEphB4 can neutralize EphrinB2-EphB4 interaction, leading to inhibition of EphB4 phosphorylation and downstream signaling [26]. Thus we examined EphB4 tyrosine phosphorylation status to evaluate the effect of sEphB4-HSA on EphB4 in tumors. As expected, sEphB4-HSA markedly reduced EphB4 phosphorylation (Figure 3B), indicating sEphB4-HSA gained access to EphB4 expressing on tumor cells and inhibited EphB4 signaling *in vivo*. Tissue analysis showed reduced vessel density (35% of control; Figure 4), cell proliferative index (23% of control; Figure 4), and increased apoptosis (4.3-fold increase over control; Figure 4). These suggest that EphB4 signaling blockade leads to inhibited tumor angiogenesis and tumor cell proliferation and also induced tumor cell apoptosis. Furthermore PI3K signaling, a major pathway downstream of EphB4 is inhibited. For example, activated or phosphorylated S6 ribosomal protein which is downstream of PI3K-Akt [31] was markedly inhibited with sEphB4-HSA treatment. We have also shown previously that VEGF and VEGFRs are expressed in 58% and 50% respectively in bladder tumor cells [32]. sEphB4-HSA has been previously shown to induce tumor VEGF [27], suggesting that combining sEphB4-HSA with VEGF inhibition may lead to enhanced anti-angiogenesis and antitumor activity. In fact, it is the case in our recent mesothelioma experiments [18]. We thus tested the combination of sEphB4-HSA and VEGF-neutralizing antibody Bevacizumab *in vivo*. When used alone, sEphB4 and Bevacizumab caused tumor volume reduction at 79% and 71% compared to control group respectively. The combination of the two however led to complete tumor regression (Figure 3). Tissue analysis also supports the combinatorial effect of sEphB4-HSA and Bevacizumab on tumor angiogensis, tumor cell growth and survival, and PI3K signaling (Figure 4).

## Discussion

This study further supports the tumor promoting role of EphB4 in TCC and highly restricted expression in tumor and not normal organ, providing opportunity for targeted therapy. We show that 32 of the 34 (94%) TCC specimens expressed EphB4. In addition, intensity of EphB4 staining in tumor specimens significantly correlated with higher tumor stage and trended to the presence of carcinoma in situ (CIS), a state of severe cellular dysplasia. In contrast EphB2 appears to be highly expressed in normal urothelium and is lost in bladder tumor. Although the exact function of EphB2 in normal urothelium is unknown, this reciprocal expression of EphB2 and EphB4 is reminiscent to what has been observed in colon and prostate cancer. In the colon, EphB2 acts to maintain undifferentiated progenitor cells in the basal crypts of the mucosa. Progressive loss of EphB2 results in lack of compartmentalization. Colon cancer cells with silencing of the tumor suppressor effects of EphB2 leads to invasive phenotype [20]. In contrast, EphB4 is overexpressed in colon cancer cells and provides survival advantage [25]. EphB2 also provides a tumor suppressor function in prostate cancer where loss of function mutation is accompanied by the increased risk for the development of prostate cancer [22]. These mutations are more common in African-American subjects who are known to have a higher risk for prostate cancer. Prostate cancer also has induction of EphB4 which provides growth and survival advantage [11], [14], [33]. Thus, accumulating evidence suggests that loss of EphB2 expression, in concert with a gain of EphB4 expression, is a common pathway towards switch from normal to tumor development and progression.

The potential clinical applicability of targeting EphB4 is exciting in both the diagnostic and therapeutic realms. EphB4 knockdown in bladder cancer cell lines with specific siRNA lead to a dose dependent decrease in cell survival. This reduction in cell viability was associated with an induction in apoptosis with a predominant activation of caspase-8 [19]. We showed previously that EphB4 facilitates bladder cancer cell migration and invasion in vitro while EphB4 specific antisense oligodeoxynucleotides inhibited growth of bladder cancer xenografts in nude mice in vivo [19]. We have since developed a novel EphB4-EphrinB2 inhibitor sEphB4-HSA, which alone induces tumor regression in human bladder cancer xenograft. sEphB4-HSA was also combined with VEGF neutralizing antibody bevacizumab based on our bladder cancer tissue analysis showing that bladder cancers express VEGFR2 at both the tumor vasculature as well as the tumor cells in some of the cases [32]. Thus targeting VEGF blocks tumor angiogenesis and in a subset of cases tumor cell directly. This rationale combination is also based on the findings that sEphB4-HSA blocks tumor angiogenesis and leads to induced hypoxia response and VEGF expression [27], [28]. Combination of sEphB4-HSA and bevacizumab induced complete regression of the tumor and thus may be worthy of investigation in humans. We have begun the clinical investigation of sEphB4-HSA in human trials. Ongoing Phase I study shows the drug is safe to administer, and efficacy studies are begun (data not shown).

Expression of EphB4 in bladder cancer also provides the opportunity to monitor urine for EphB4-expressing cancer cells, a potential use as a tumor marker for diagnosis, prognosis and response. Furthermore antibody to EphB4 conjugated with cytotoxic agents may also be applied for local and systemic therapy. Thus, EphB4 provides new mechanistic insights and novel diagnostic and therapeutic opportunities for bladder cancer.

## Supporting Information

File S1 **Figure S1 & S2 and Table S1 & S2.** Figure S1. Representative EphB4/EphB2 immunostaining on tumor and normal urothelium obtained from the same patient during cystectomy. 2 paired cases are shown. Nuclei were counter-stained with DAPI. Figure S2. EphB4 siRNA knockdown induces apoptosis in bladder cancer cell but not normal bladder cell. 5637 and PD071 cells were grown in 6-well plate and transfected with 50 nM EphB2 or EphB4 siRNA with Lipofectamine 2000 (Invitrogen). 48 hours later, cells were harvested with Cell Dissociation Buffer (Sigma), stained with Annexin V apoptosis quantitation kit (Biotium), and analyzed on flow cytometer LSRII (BD Biosciences). Dead cells were excluded by 7-AAD staining. Data were analyzed with FlowJo (Tree Star). A, EphB2 siRNA had no effect on apoptosis of 5637, whereas EphB4 siRNA led to significant apoptosis. B, Both EphB2 and EphB4 siRNA had no effect on PD071 apoptosis.
(PPTX)
