# Supplementary material for: The Differential Expression of EphB2 and EphB4 Receptor Kinases in Normal Bladder and in Transitional Cell Carcinoma of the Bladder
Source: PLoS One. 2014 Aug 22;9(8):e105326. doi: 10.1371/journal.pone.0105326 (PMC4141800; doi:10.1371/journal.pone.0105326)
Supplement: File S1 — Figure S1 & S2 and Table S1 & S2. Figure S1. Representative EphB4/EphB2 immunostaining on tumor and normal urothelium obtained from the same patient during cystectomy. 2 paired cases are shown. Nuclei were counter-stained with DAPI. Figure S2. EphB4 siRNA knockdown induces apoptosis in bladder cancer cell but not normal bladder cell. 5637 and PD071 cells were grown in 6-well plate and transfected with 50 nM EphB2 or EphB4 siRNA with Lipofectamine 2000 (Invitrogen). 48 hours later, cells were harvested with Cell Dissociation Buffer (Sigma), stained with Annexin V apoptosis quantitation kit (Biotium), and analyzed on flow cytometer LSRII (BD Biosciences). Dead cells were excluded by 7-AAD staining. Data were analyzed with FlowJo (Tree Star). A, EphB2 siRNA had no effect on apoptosis of 5637, whereas EphB4 siRNA led to significant apoptosis. B, Both EphB2 and EphB4 siRNA had no effect on PD071 apoptosis. (PPTX) [file pone.0105326.s001.pptx]

## Slide 1
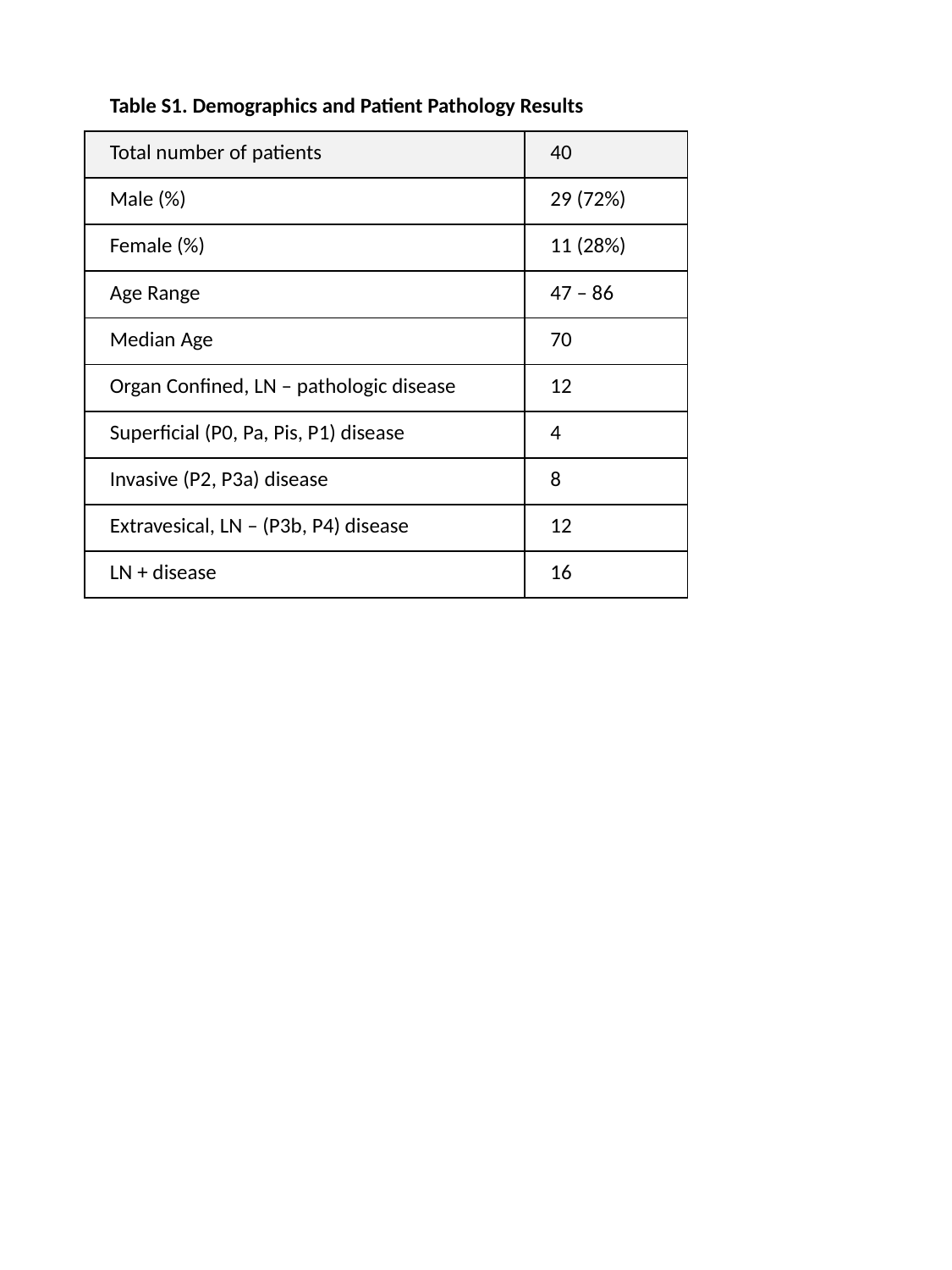

| Table S1. Demographics and Patient Pathology Results | |
| --- | --- |
| Total number of patients | 40 |
| Male (%) | 29 (72%) |
| Female (%) | 11 (28%) |
| Age Range | 47 – 86 |
| Median Age | 70 |
| Organ Confined, LN – pathologic disease | 12 |
| Superficial (P0, Pa, Pis, P1) disease | 4 |
| Invasive (P2, P3a) disease | 8 |
| Extravesical, LN – (P3b, P4) disease | 12 |
| LN + disease | 16 |

## Slide 2
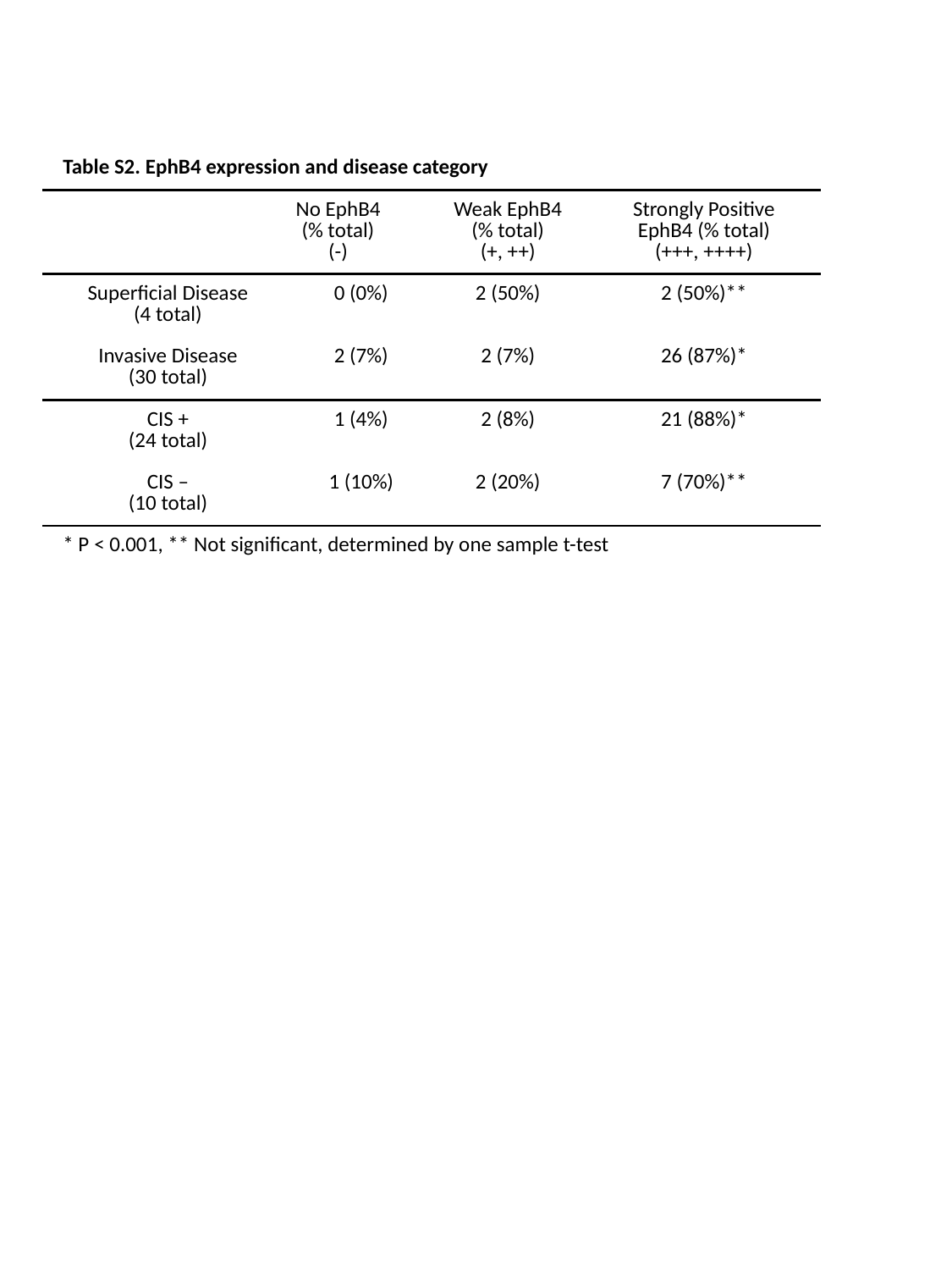

| Table S2. EphB4 expression and disease category | | | | |
| --- | --- | --- | --- | --- |
| | No EphB4 (% total) (-) | | Weak EphB4 (% total) (+, ++) | Strongly Positive EphB4 (% total) (+++, ++++) |
| Superficial Disease (4 total) | | 0 (0%) | 2 (50%) | 2 (50%)\*\* |
| Invasive Disease (30 total) | | 2 (7%) | 2 (7%) | 26 (87%)\* |
| CIS + (24 total) | | 1 (4%) | 2 (8%) | 21 (88%)\* |
| CIS – (10 total) | | 1 (10%) | 2 (20%) | 7 (70%)\*\* |
| \* P < 0.001, \*\* Not significant, determined by one sample t-test | | | | |

## Slide 3
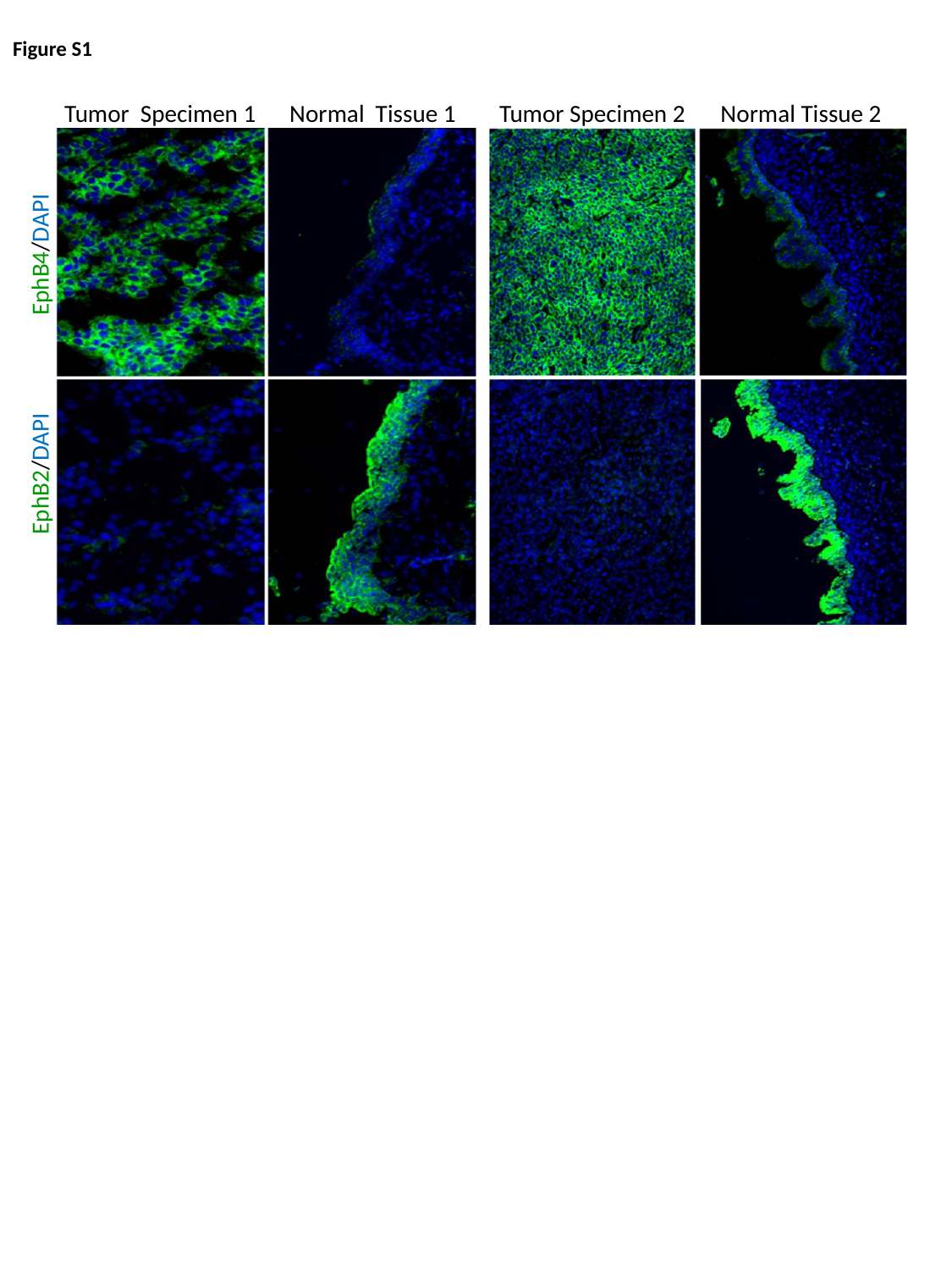

Tumor Specimen 1
Normal Tissue 1
Normal Tissue 2
 Tumor Specimen 2
Figure S1
EphB4/DAPI
EphB2/DAPI

## Slide 4
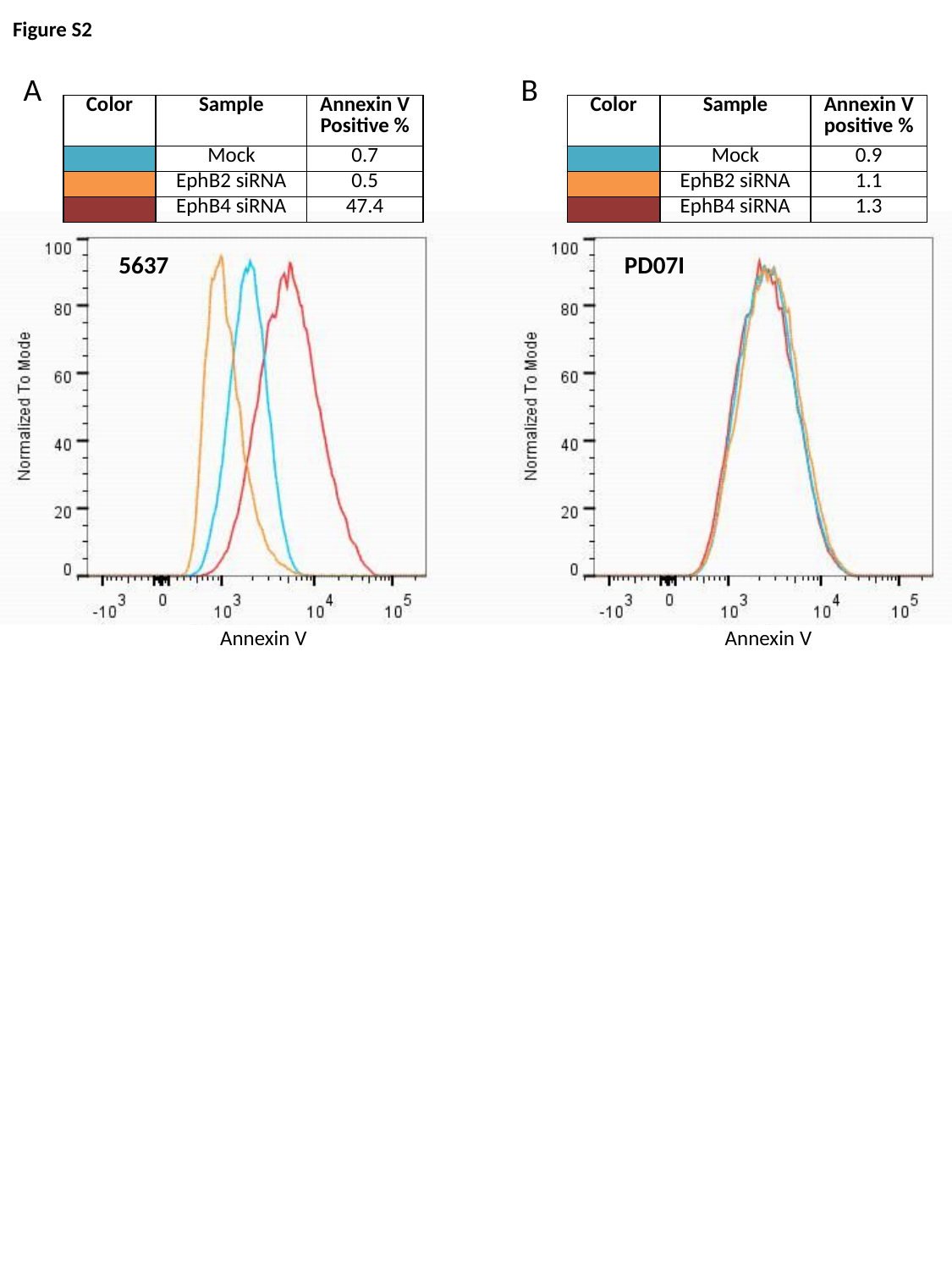

Figure S2
A
B
| Color | Sample | Annexin V Positive % |
| --- | --- | --- |
| | Mock | 0.7 |
| | EphB2 siRNA | 0.5 |
| | EphB4 siRNA | 47.4 |
| Color | Sample | Annexin V positive % |
| --- | --- | --- |
| | Mock | 0.9 |
| | EphB2 siRNA | 1.1 |
| | EphB4 siRNA | 1.3 |
5637
PD07I
Annexin V
Annexin V
